# The Decreased Complexity of Blood Pressure Dynamics Is Associated With Higher White Matter Lesions in Older Adults

**DOI:** 10.1093/geroni/igaa057.687

**Published:** 2020-12-16

**Authors:** Xin Jiang, Xia Gao, Hui Zhang, Wuhong Deng, Wen Fu, Brad Manor, Lewis Lipsitz, Junhong Zhou

**Affiliations:** 1 Shenzhen People’s Hospital, Shenzhen, Guangdong, China; 2 Shenzhen People’s Hospital, Shenzhen, China; 3 Hebrew SeniorLife/Harvard Medical School, Boston, Massachusetts, United States; 4 Hebrew SeniorLife and Harvard Medical School, Boston, Massachusetts, United States; 5 Harvard Medical School/Hebrew SeniorLife, Roslindale, Massachusetts, United States

## Abstract

White matter lesions (WML) are highly prevalent in older adults and thought to represent cerebral microvascular disease, contributing to slow gait and dementia. Hypertension is associated with WML. However, the underlying mechanism of this association is unclear. The complex beat-to-beat BP fluctuations represent the influence of BP regulatory mechanisms over multiple time scales. The association between WML and abnormalities in BP regulation may be manifest as a loss of complexity in BP dynamics. The aim of this study is thus to explore the relationships between hypertension, BP complexity, and WML in older adults. Twenty-two older adults with hypertension (SBP>140 mmHg) and 19 age-matched older adults without hypertension (i.e., control) completed this study. Their whole-brain WML were assessed by two neurologists using the Fazekas Scale. Greater score reflects higher WML grade. Each participant completed a 10-minute BP assessment when sitting quietly following the MRI. The continuous SBP and DBP series were recorded, and the complexity of them was quantified using multiscale entropy (MSE). Lower MSE reflects lower complexity. Compared to the controls, hypertensives had significantly greater Fazekas scores (i.e., higher WML grade) (F=4.8, p=0.02) and lower complexity of SBP and DBP (F>3.7, p<0.01), after adjusting for age. Across two cohorts, those with lower SBP and DBP complexity had higher Fazekas score (r<-0.51, p<0.01), and this association was independent of age and group. These results suggest that WML are associated with a loss of complexity in BP dynamics. Future longitudinal studies are needed to examine the causal relationship between WML and BP.

